# Seasonality and Strain Specificity Drive Rapid Co-evolution in an *Ostreococcus*-Virus System from the Western Baltic Sea

**DOI:** 10.1007/s00248-023-02243-5

**Published:** 2023-06-03

**Authors:** Luisa Listmann, Carina Peters, Janina Rahlff, Sarah P. Esser, C-Elisa Schaum

**Affiliations:** 1 Institute for Marine Ecosystem and Fisheries Science, University of Hamburg, Olbersweg 24, 22767 Hamburg, Germany; 2 Centre for Earth System Science and Sustainability, 20146 Hamburg, Germany; 3 Group for Aquatic Microbial Ecology, Environmental Microbiology and Biotechnology, Departement of Chemistry, University of Duisburg-Essen, 45141 Essen, Germany; 4 Present Address: Centre for Ecology and Evolution in Microbial Model Systems (EEMiS), Department of Biology and Environmental Science, Linnaeus University, 39231 Kalmar, Sweden; 5 Environmental Metagenomics, Research Center One Health Ruhr of the University Alliance Ruhr, Faculty of Chemistry, University of Duisburg-Essen, 45141 Essen, Germany

**Keywords:** Phytoplankton, Virus, Cross-infection, Co-evolution, Evolutionary history

## Abstract

Marine viruses are a major driver of phytoplankton mortality and thereby influence biogeochemical cycling of carbon and other nutrients. Phytoplankton-targeting viruses are important components of ecosystem dynamics, but broad-scale experimental investigations of host-virus interactions remain scarce. Here, we investigated in detail a picophytoplankton (size 1 µm) host’s responses to infections by species-specific viruses from distinct geographical regions and different sampling seasons. Specifically, we used *Ostreococcus tauri* and *O. mediterraneus* and their viruses (size ca. 100 nm). *Ostreococcus* sp. is globally distributed and, like other picoplankton species, play an important role in coastal ecosystems at certain times of the year. Further, *Ostreococcus* sp. is a model organism, and the *Ostreococcus*-virus system is well-known in marine biology. However, only few studies have researched its evolutionary biology and the implications thereof for ecosystem dynamics. The *Ostreococcus* strains used here stem from different regions of the Southwestern Baltic Sea that vary in salinity and temperature and were obtained during several cruises spanning different sampling seasons. Using an experimental cross-infection set-up, we explicitly confirm species and strain specificity in *Ostreococcus* sp. from the Baltic Sea. Moreover, we found that the timing of virus-host co-existence was a driver of infection patterns as well. In combination, these findings prove that host-virus co-evolution can be rapid in natural systems.

**Supplementary Information:**

The online version contains supplementary material available at 10.1007/s00248-023-02243-5.

## Introduction

Phytoplankton viruses and hosts are omnipresent in the marine environment. Within the microbial loop, viruses can infect both hetero- and autotrophic microbes and “shunt” carbon away from the classical food web [1, 2]. Consequently, viruses can have far-reaching effects on biogeochemical cycles, and in phytoplankton communities, viruses exert strong top-down control [3]. The biology and diversity of phytoplankton viruses from natural assemblages are well-studied via independent genomic analyses [4]. These analyses are now facilitated because prediction and binning tools (genomic analyses) for eukaryotic viruses have significantly advanced [5–7]. However, the genetic analyses of natural assemblages alone are not enough to understand underlying ecological processes and consequences of viral infections in phytoplankton. Therefore, cultivation and experimental approaches in the laboratory are crucial tools [8].

The ecology of viruses hinges on biotic (e.g. specificity of infections) and abiotic (the environmental conditions under which infections happen) aspects. Thus, we need to understand how strong and specific infections are and how often and under what conditions they occur. Seasonal changes that affect sea surface temperature, salinity, and nutrient conditions might be particularly relevant as shown for Baltic Sea phages of Gammaproteobacteria [9] and *Ostreococcus* viruses [4]. This is because phytoplankton host abundance, and diversity can also change on seasonal timescales [10]. Where biotic aspects of viral ecology have been studied, infections were highly species or even strain specific [11, 12], and diversity within species had the potential to affect the host range of infections. Only a handful of studies have investigated the effects of abiotic factors [13, 14]. Baltic Sea oceanography and ecosystems are very unique but changing at an unprecedented pace; hence, a better understanding of the baseline interactions between viral infection dynamics and environmental conditions on a temporal-spatial scale is crucial.

At a size of about 1 µm, *Ostreococcus* species complex are the smallest known free-living photosynthetic eukaryotes [15]. *Ostreococcus* spp. are ecologically relevant model organisms due to their global distribution and prevalence in many marine environments [16]. They are infected by large DNA viruses (ca. 100 nm) of the phylum Nucleocytoviricota. Like their hosts, the viruses have been found globally [17] including different *Ostreococcus* virus species in the pelagic of the Baltic Sea [18]. The known members of the *Ostreococcus*-virus system are lytic with the potential for rapid development of host resistance. This has been described in several recent studies that also investigated genetic mechanisms of the dynamics [11, 19]. In addition, a case of virion producing resistant host cells has been described, indicating the potential of lysogeny within this system [20]. The aforementioned studies have focused on a confined geographical region in the Mediterranean Sea and/or time of year but did not investigate how infectability and resistance patterns can change within a few generations (i.e. weeks to months).

Here, we investigated the biology of different *Ostreococcus*-virus systems freshly isolated from different regions of the Baltic Sea by studying the infection patterns of both hosts and viruses that have experienced different evolutionary histories. The Baltic Sea allows investigating environmental gradients such as North-east to South-west salinity and temperature gradients and their variability [21] within geographical regions that are in proximity to mitigate confounding effects, such as light and nutrient availability. In the future, the Baltic Sea will become both sweeter and warmer, meaning that comparing different conditions present today will provide important insights into potential future perturbations [22]. Within the *Ostreococcus*-virus system from the Baltic Sea, we are particularly interested in the following questions: (i) When and where are *Ostreococcus* and its viruses likely present? (ii) How species or strain specific are infections in the Baltic Sea *Ostreococcus*-virus system? and (iii) How do infection patterns change over time or between geographical regions?

## Material and Methods

### Ostreococcus and Virus Isolations

**Fig. 1:**
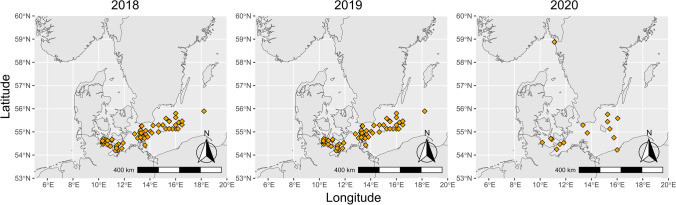
The three panels show the stations in the Western Baltic Sea that were sampled during 3 consecutive years. The sampling effort in 2019 was the highest with five cruises in total

In addition to previously isolated and studied strains of *Ostreococcus* [23], we isolated further 20 novel strains of *Ostreococcus mediterraneus* and 79 new viral strains. During seven R/V cruises from 2018 to 2020 (AL505, AL513, AL520, AL521, AL522, AL524, AL530, and Assemble Plus project MIDSEAS), we collected water samples at 105 stations (Fig. 1) in the Western Baltic Sea. The regions of the Baltic Sea included 4 different areas: the coastal Swedish Skagerrak (SW) close to Tjärnö Marine Station, the Kiel area (KB and MB, Kiel Bight, and Mecklenburg Basin, respectively), the Arkona Basin (AK), and Bornholm Basin (BB).

The water samples were taken at depths between 1 and 5 m and immediately filtered through a 35-µm mesh (Hydrobios®) to remove grazers. Further 2 L of two size fractions each were concentrated to increase the biomass within a sample: First, 2 L of 2 µm filtrate were concentrated to 200 mL for later *Ostreococcus* isolation. Second, 2 L of filtrate from 0.45-µm pore size filter (Whatman®) were concentrated using tangential flow filtration (100,000 MWCO PES Sartorius®). Before continuing with either *Ostreococcus* or virus isolations, the samples were analysed via flow cytometry to check for the prevalence of host or viral populations in the sample (see SI for flow cytometry description).

*Ostreococcus* spp. were isolated using the isolation by dilution method [24]. For the isolation by dilution, samples were diluted to < 1 cell per specific volume and grown in 48-well plates for ca. 15–30 days until greening was detected. Possible *Ostreococcus* cultures were kept at their respective isolation salinities in f/2 media that was prepared using artificial seawater (further information SI). The cultures were kept at 18 °C and 12:12 day and night cycles at 150-µE light intensity. Upon successful identification of *Ostreococcus* strains, viruses were isolated the following way: 20 mL of each *Ostreococcus* strain (0.5–2 *10^6^ cells L^−1^) was combined with 1 mL of the 0.45-µm concentrated water sample of the respective geographical area (see Fig. S3; all combinations of hosts and virus water samples in upper panel, grey boxes) resulting in ~ 250 combinations. After 5, 10, and 14 days, the cell numbers were counted via flow cytometry, and any *Ostreococcus* culture that had ≤ 50% cells than a control was considered lysed (Fig. S3, green boxes in upper panel). These cultures were then filtered through a 0.45-µm filter membrane (Whatman®), and the lysate was used for a second round of liquid infection. After a second successful infection, the lysate was used in two rounds of plaque formation to isolate single virus strains. Specifically, 100 µL of diluted lysate was added to a culture of *Ostreococcus* (2 *10^6^ cells mL^−1^), and then agarose (Thermofisher®) was added to a final concentration of 0.2%. After growth in a petri dish for up to 14 days, single plaques (clearings on the *Ostreococcus* lawn) formed and could be picked for the production of larger volumes of lysate. Lysates were always stored at 4 °C. Two months before the cross-infection experiment was started, 40 mL of fresh lysate of all virus strains was prepared.

It is important to mention that the isolation procedures we chose here, select for the fastest growing *Ostreococcus* hosts as well as the most infective viruses in these host-virus systems. As a consequence, the full ecologically relevant diversity may not be covered by this approach.

### Species Identity and Diversity

For DNA extraction, 2 mL of exponentially growing *Ostreococcus* culture was pelleted via centrifugation at 8000 g. The pellet was then used for DNA extraction with the PROMEGA© ReliaPrep gDNA Tissue Miniprep system (following the standard protocol for animal tissue) and yielded DNA in the range of 5–80 ng µL^−1^. For species identification, we used the 18S rRNA gene sequence (Primers 18S F 5′-ACCTGGTTGATCCTGCCAG-3′ and 18S R 5′-TGATCCTTCCGCAGGTTCAC-3′; 60 °C [25]) and PCR chemicals (GoTaq PROMEGA©) with the following PCR conditions: 2 min at 95 °C, 35 cycles with 30 s at 95 °C – 1 min at 60 °C – 30 s at 72 °C.

Viral particles were prepared by centrifuging 2 mL of lysate at maximum speed (15,000 g) for 10 min. The supernatant was discarded and viral particles suspended in 30 µL Tris–EDTA buffer. We used primers for the DNA polymerase B gene (polB) (VpolAS4 GARGG[I]GC[I]AC[I]GT[I]YTNGA and VpolAAS1 CC[I]GTRAA[I]CCRTA[I]AC[I] SWRTTCAT) [26]. The same PCR chemicals as for the 18S rRNA gene were used, but the PCR conditions were 2 min at 95 °C, 35 cycles with 30 s at 95 °C – 1 min at 60 °C – 30 s at 72 °C.

All PCR products were cleaned up with a kit (Fisher Scientific PCR cleanup®) prior to Sanger sequencing using the forward primer at EUROFINSGENOMICS®. The 18S rRNA gene sequences were clipped and trimmed for further analysis (sequence length after trimming 551 bp), and taxonomic identification was performed with Codon Code Aligner (Version 9.0.1) and the following reference used: reference genome from [27], GenBank CAID01000001.2 to CAID01000020.2, RCC2590_scf16, BCC102-466,838, Rcc809_18S_fromgenome, ostta12g00750, and Olu_18S_fromgenome. The polB sequences were also clipped and trimmed (sequence length after trimming 281 bp) for further analysis, but here we then used the NCBI BLAST search tool [28] for species identification. The default settings for “MEGA-BLAST” search were chosen. Results for the *e*-value were at least < 6e-50, percent identity > 90% and the coverage > 90%.

Both 18S rRNA and polB gene sequences (55 yielded clean sequences) were then used to build neighbour-joining phylogenetic trees (Codon Code Aligner) to identify relatedness. Default settings of the software for neighbour-joining trees based on number of base-pair differences between sequences were used and missing data considered as deletions. The calculated trees were then exported as newick format and subsequently visualized using the package “ggtree” in R version 4.2.2 [29]. We used the sequencing results to compare to the infection patterns.

In addition to the species identity, we added the information of the water sample’s origin (geography and season) to the names of hosts and viruses. Since viruses have an inherent host dependency, they were also named in relation to the water sample of their hosts. This additional information was used to analyse the infection patterns in the following experiment.

### Cross-Infection Experiments

In total, 17 *Ostreococcus* sp. strains (2 *O. tauri* and 15 *O. mediterraneus*) were infected by all 79 different *Ostreococcus* viruses. Specifically, we prepared four petri dishes with *Ostreococcus* culture in semi-solid 0.2% agarose (Thermofisher®) and added 16 virus strains per petri dish. For each virus strain, we pipetted 2 × 2 µL of lysate onto the solidified culture. The multiplicity of infection (MOI) of all infections was 10:1 where the *Ostreococcus* cultures had a starting concentration of 5*10^6^ cells mL^−1^ and the virus lysates contained 50*10^6^ viral particles mL^−1^ (see Fig. S1 for petri dish schematic). The petri dishes were kept at 18 °C and a 12:12 day and night cycle at 150 µE light intensity.

After 3, 5, 7, and 10 days of incubation, we recorded plaque formation (presence/absence) and took photographs of the petri dishes using a Canon digital camera (EOS M100). Plaque size was measured via ImageJ® (V 1.53t) [30], and the rate of plaque size increase in mm day^−1^ was calculated based on the slope of a linear regression fitted to the size data from days 3 to day 10 post infection.

### Statistical Analysis

We used the bipartite network analysis to test for nestedness in the infection patterns using ‘computeMod()’ in the bipartite package in the R programming environment [31]. To test the significance of the patterns, we calculated the nestedness metric based on overlap and decreasing fill (NODF) of 1000 null models and compared their distribution with the NODF of the observed matrix.

Next, we analysed the rate of plaque size increase for the infections. Due to non-normalized data, we used Kruskal–Wallis-Tests and subsequent pairwise Wilcoxon-Test with Bonferroni corrections. The following factors were tested: clusters, virus seasonal and geographical origin, host seasonal and geographical origin, and difference between the infection on the isolation and a foreign host. All comparisons were then plotted in boxplots.

All distance matrix calculations as well as statistical analyses were carried out using R software (R Version 4.2.2. [29]) and the following specific packages in the R environment: ggplot2, gridExtra, ggpubr, reshape2, nlme, rnaturalearth, bipartite, and ggtree.

## Results

### Isolation Success

**Fig. 2:**
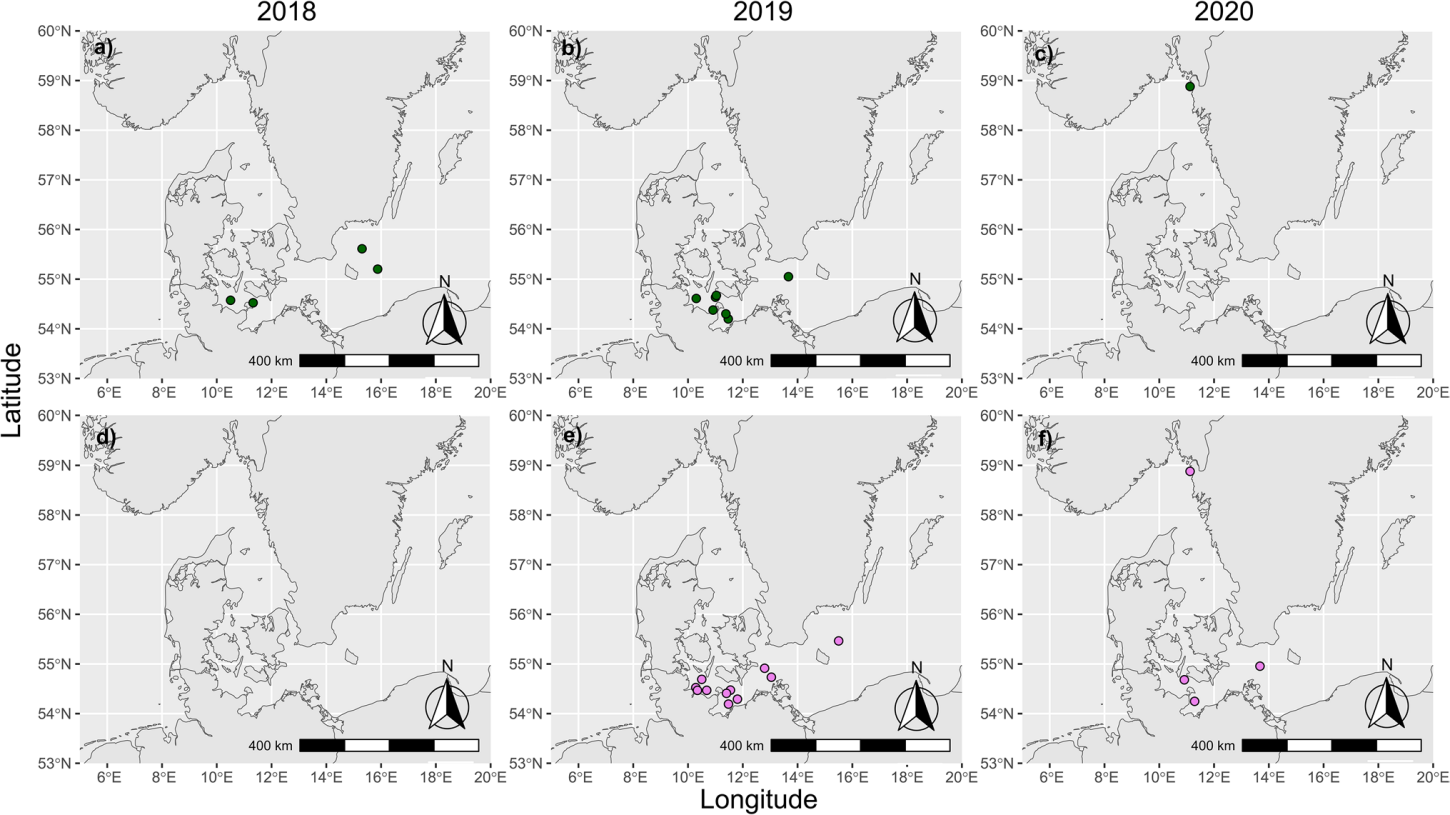
The panels show all stations from which we successfully isolated *Ostreococcus* sp. (green points in panels **a**–**c**) and their viruses (violet points in panels **d**–**f**). In the year 2018, no water samples were collected for virus isolations. Note that we used *Ostreococcus* hosts in combination with all water samples from the same year to start the isolation procedure of viruses

Out of 119 water samples, we successfully isolated two *Ostreococcus tauri* and 19 *Ostreococcus mediterraneus* strains. These strains originated largely from KB and MB, with a higher isolation success during spring and autumn phytoplankton blooms. The isolation success was approximately 7% as we isolated *Ostreococcus* strains from 8 out of 119 water samples. Then, we were able to isolate 9 *Ostreococcus tauri* viruses (OtVs) and 70 *Ostreococcus mediterraneus* viruses (OmVs) from 13 of the *Ostreococcus* strains using 84 different water samples (Fig. S3 overview of virus isolations). The viruses mainly originated from the KB and MB in spring and early summer (March 2018 to May 2020) (Fig. 2).

**Fig. 3:**
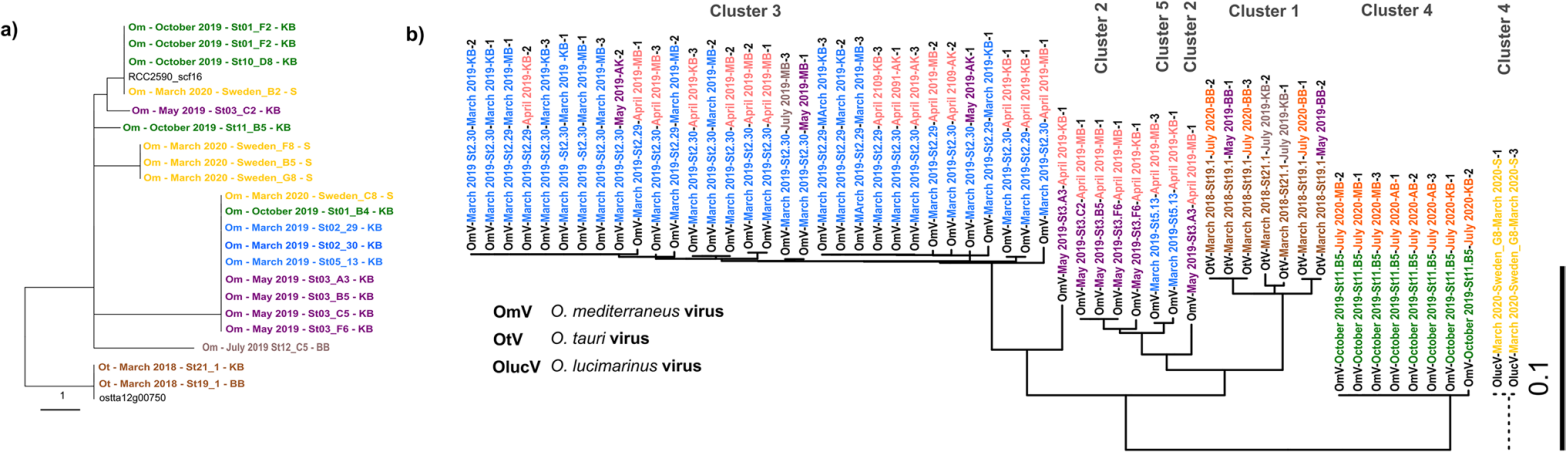
Panels **a** and **b** show the genetic trees of the *Ostreococcus* and virus strains based on 18S rRNA gene (551 bp) and DNA polymerase beta (PolB) sequences (241 bp), respectively. Each of the virus strain names indicates the virus species, the isolation host, the seasonal and geographic origin of the water sample, and the number of the viral strain of that combination (e.g. OmV-May 2019_St.3_A3-March 2019-MB-2). The colours indicate the different seasons from when the water samples were taken. Two- and one-letter abbreviations at the end of the names indicate the geographical region where the water samples for virus isolations were taken (KB, Kiel Bight; MB, Mecklenburg Bight; AB, Arkona Basin; BB, Bornholm Basin; S, Sweden). The sequences of OlucV were too different to be aligned with the rest of the PolB sequences. The scale bars indicate evolutionary distance based on ggtree calculation

The 18S rRNA gene analysis (based on nucleotide sequence) identified the host strains to be *O. tauri* and *O. mediterraneaus* with single nucleotide genetic differences as previously described in Subirana et al. [32] (Fig. 3a). The two *O. tauri* strains are grouped in the lower branch of the tree, whereas the *O. mediterraneus* strains are grouped in the upper branches of the tree. The polB nucleotide sequence analysis of the viruses with NCBI Blast revealed three different virus species (Fig. 3b, O*. mediterraneus* virus (OmV), *O. tauri* virus (OtV), and *O. lucimarinus* virus (OlucV) in names). Note that even though the host of OlucV virus was *O. mediterraneus*, the polB sequence was most similar to that of an *O. lucimarinus* virus. Compared to the 18S rRNA gene sequences, we found more nucleotide variability among the viruses forming five strain clusters (Fig. 3b).

### Cross-Infection Experiment

**Fig. 4:**
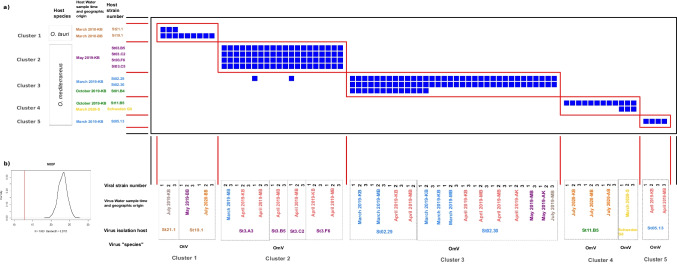
Panel **a** shows the infection matrix after the modularity analysis (note that only strains where infection was successful are shown in the matrix). The virus strains are shown on the x-axis, whereas the host strains are shown on the y-axis. The colours on the axes’ labels indicate the timing of the cruises from which the hosts were isolated or from which the water samples of the viruses were collected. Blue squares framed with red lines indicate successful infection clusters. Panel **b** shows the results of the NODF distribution of the calculated null models and the observed NODF (red line) indicating that the depicted modularity of the infection matrix was significant

Of the 1343 cross-infection combinations, we found 206 successful infections in 13 of the 17 *Ostreococcus* strains (Fig. 4a, blue squares in the infection matrix). These successful infections were clustered into five modules (clusters 1–5) characterized by species and/or seasonal specificity (Fig. 3b). In clusters 2 and 3, overall, viruses isolated from hosts from the same time of the year were grouped together. However, we noticed a difference in host ranges between the viral clusters: In cluster 2, all viruses isolated on May 2019 hosts infected all hosts from that time. In contrast, in cluster 3, all viruses from two March 2019 hosts were grouped together, but some viruses also infected a host from October 2019, and none infected the other host from March 2019 (Om-March 2019-St05.13). In clusters 1, 4, and 5, we found that the viruses were mainly host specific, with the exception of the viruses of host 21.1 and Sweden G8 that also infected hosts that were regionally far away. It is important to indicate that the modularity patterns relate to the viruses’ host origin, but the virus water samples came from different times. For example, the difference between clusters 2 and 1 is that the virus water samples in cluster 2 were from the same season (May 2019 vs. April 2019), whereas in cluster 1, the virus water samples were from 1 or 2 years later (March 2018 vs. May, July 2019, and July 2020). However, these differences in the virus’ seasonal origins did not affect the modularity pattern.

**Fig. 5:**
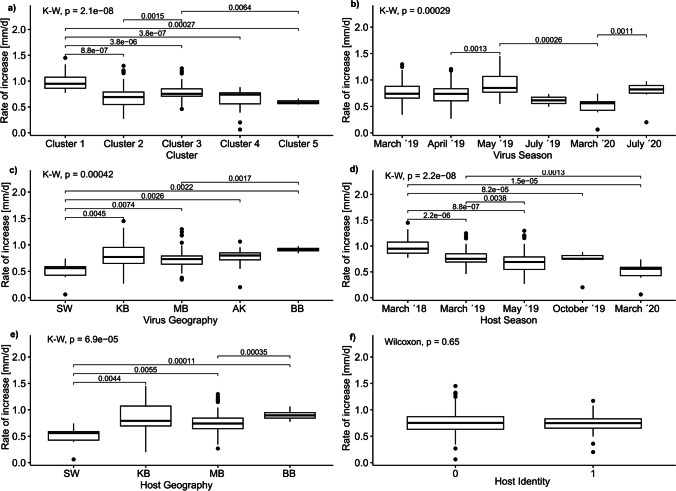
The figure shows the rate of increase in plaque sizes of the successful infections. In each panel, the result of the Kruskal–Wallis test for the tested factors (noted on the x-axis) is shown at the top left. The bars between the groups indicate significant differences based on pairwise Wilcoxon tests. Each panel shows a different factor that was analysed, starting with the differences between clusters (panel **a**), then how the plaque size increase varies when considering the origin of the virus water samples in panels **b** and **c**, and finally comparing the plaque size increase between the origin of the host water samples in panel **d** and **e**. Panel **f** shows the difference in plaque size increase in infections on known (0) and foreign hosts (1). The boxplots indicate the median and upper and lower quartile with lines showing the maximum range. Dots indicate outliers in the groups

The infection plaques appeared in most cases on day 3 post infection, whereas some only appeared on the 5th or 7th day post infection (Fig. S4). At the same time, the plaques grew with different velocities (Fig. S4 slope of regression lines) from 0.2 to 1.5 mm day^−1^. From the first to the last cluster, the plaques grew slower (Fig. 5a, Chi^2^ = Clusters: _4_ = 41.506, *p* < 0.001). Similarly, there was a tendency for slower plaque growth for hosts that were isolated at a later time (Fig. 5d, Chi^2^ = Host Season: _4_ = 41.455, *p* < 0.001). The opposite was the case when comparing the geographical origins of the water samples: the further to the east the water samples were taken (SW to BB), the faster the plaques grew (Fig. 5 c and e, Chi^2^ = Virus Geography: _4_ = 20.356, *p* < 0.001, Chi^2^ = Host Geography: _3_ = 21.883, *p* < 0.001). The seasonal origin of the virus water sample also had an effect on how fast plaques formed, but no specific time trend could be found (Fig. 5b, Chi^2^ = Virus Season: _4_ = 23.337, *p* < 0.001).

## Discussion

Over a 2-year sampling period, we successfully isolated 21 hosts and 79 virus strains of the *Ostreococcus* sp. complex from different regions of the Baltic Sea as well as the Swedish Skagerrak and investigated infection patterns within this system. We identified the evolutionary history, and thus the timing of when hosts and their associated viruses coexisted, as the main driver of infection patterns. In addition, host species and strain specificity underline the present understanding of rapid host-virus co-evolution [33, 34], and here, we demonstrate this in a phytoplankton-virus system that evolved in natural conditions.

Based on metagenomic analyses, *Ostreococcus* sp. is present all year round in the Baltic Sea, but its prevalence varies throughout the year and regionally [4]. This is in line with the isolation success we had over the 2-year sampling period, where we were mainly able to isolate *Ostreococcus* strains in spring (March to May) and autumn (October). Sampling success of our focal species likely depends on the composition of the whole phytoplankton community at time of sampling: Spring blooms in the Western Baltic Sea are dominated by large and often chain-forming diatoms [21] that can be easily separated via sieving from the smaller picoplankton fraction. When applying nutrient enrichment excluding silicate and growth at lower (ca. 15 °C) temperatures, *Ostreococcus* sp. is favoured over other picoplankton species and diatoms for isolation approaches. In contrast, during summer blooms, the Western Baltic Sea phytoplankton community is dominated by cyanobacteria [35]. These cyanobacteria are the dominant picoplankton species in summer and hinder the isolation success of *Ostreococcus* sp. in this season [36]. Our findings on timely biases for isolation success have also been observed in the Mediterranean *Ostreococcus* populations that are mainly present in early times of the year in the Mediterranean Sea. Based on molecular evidence, *Ostreococcus* and its viruses were found even in the eastern part of the Baltic Sea [4] but at the lowest relative frequencies compared to higher salinity regions Similarly, our success of isolating *Ostreococcus* was also higher in the Western sampling areas with higher salinities. As *Ostreococcus* are primarily a marine species, the pronounced brackish conditions in the Eastern Baltic Sea could hinder prevalence of algae in that region [37]. We conclude that for the successful isolation of phytoplankton species, it is crucial to know when the species of interest is likely to be either dominant in the community or easily separable from other community members.

The 18S rRNA gene sequences revealed that our *Ostreococcus* species belonged mainly to the subclade D, also named *O. mediterraneus*, while two strains belonged to the *O. tauri* clade C [16, 32]. This is surprising because, for example, Zeigler et al. [4] found that most of the *Ostreococcus* sequences were identified as *O. tauri*. Within the species complex of *Ostreococcus*, there are very few base pair differences within the 18S rRNA gene [32], and since *O. tauri* was the first species to be isolated and identified within this picoplankton species complex [38], available genomic data could actually underestimate the prevalence of each of the four subclades A–D [39].

The genetic relatedness of the isolated virus strains based on Pol B sequences (typically used gene for identification and phylogenetic classification [40, 41]) showed a clustering into groups based on the host’s origin and the infection clusters. This is in line with a previous study showing that genetically related viruses tend to infect the same hosts or group of hosts [11]. The pol B sequence of the two viral strains originating from the Sweden_G8 *O. mediterraneus* host did not align with the rest of the sequences because they were more similar to *O. lucimarinus* pol B sequences based on BLAST comparisons. This was a contradictory result [42] because following the “identification” of the virus based on its host origin (ecological identification), we would have had to call the virus an *O. mediterraneus* virus. Therefore, the genetic identification of viruses may not be adequate to deduce its host origin or its role in the ecosystem. We will now further discuss the ecology of the infections in this study considering the viruses host origin.

In previous studies, different virus or host characteristics have been described, ranging from generalist to specialist viruses or highly susceptible to highly resistant hosts. Infection patterns based on the virus or host characteristics have been described in [43]. Clusters of infection can form when a set of hosts or viruses exhibit the same traits with regard to infectability or resistance. Identifying what drives modularity in infection networks is key to understanding the ecology of the infection dynamics in a system. In our study, we found that the modules in the infection patterns were not random (see Fig. 3, panel b). Some viral clusters could be associated to the isolation time of the host, and some infections were strain-specific, but all except one were species-specific. Specifically, for the clusters associated to seasons, we found that the viruses that were isolated from hosts from a specific time of year (March or May) were also able to infect hosts from that same time. However, all other hosts that were tested were not infected. The strain/species specificity underlines previous findings on host specificity in the *Ostreococcus* virus system [11]. The seasonal modularity, however, indicates co-evolutionary patterns in this host-virus system on a timescale that has previously been described and studied in another phytoplankton-virus system [11, 33, 34].

While it is known that viruses are species-specific, infections can also be specific to environmental conditions [44]. We may therefore find that viruses are specifically able to infect only hosts from similar seasons, for example, when sea surface temperatures stay within a specific range (see, for example, clusters 3 and 4, and see Fig. S5 for changes in abiotic environment over time). In addition to regular saltwater inflow from the North Sea, the Baltic Sea experiences an increasing inflow of water from coastal freshwater sources (related to precipitation and in spring after snow melt). This induces stratification [45], which in turn leads to increasing temperatures in upper water layers. These conditions might also allow *Ostreococcus* spp*.* to flourish [46]. Being able to cross-infect in spring/March and independent of changes in salinities could be an important asset for viruses of *Ostreococcus* to take advantage of thriving subpopulations of the host.

*Ostreococcus* spp. are non-blooming picoplankton species that are adapted to the environmental conditions (such as temperature, salinity, and nutrients) that they have experienced in the past (i.e. several 10 s to hundreds of generations, see Listmann et al. [47]). These adaptations do not only relate to physiological or fitness-related traits but likely also the biotic interactions with viruses. Between the seasons from which the hosts were isolated and which related to the infection modules, the temperature and sometimes salinity but also light conditions varied. For instance, day-to-day salinity variations in the upper few centimetres of the water column during the sampling campaign in the Swedish Skagerrak ranged from 22.5 to 30.0 (supplement of Rahlff et al. [48]). The virus OlucV_March 2020_Sweden_G8_March_2020_S3 isolated from a water sample from this station was still capable of infecting hosts from the Western Baltic Sea, which are usually subjected to much lower salinities (Fig S6 panel a vs. panel b; Western vs. Eastern sampling area). Another example of viruses from a higher salinity environment that were able to infect a host from a lower salinity environment is OtV_March 2018_St21.1_July 2019_KB. These examples could reflect the viruses’ adaptation to salinity gradients, which seemingly did not negatively impact host range and infection capabilities. In line with that, a study by Bellec et al. [49] showed that coastal marine lagoons, which due to their shallowness are also more prone to salinity changes, contained *O. tauri* viruses that were also abundant in offshore and coastal ecosystems.

Further infection experiments with changes in salinity or temperature as compared to pure co-evolutionary studies in this system would shed light on these two possible explanations of the infection patterns we found. In addition, finding differences in infection patterns depending on the environmental condition could allow us to make predictions on how phytoplankton would cope under future climate conditions.

The variations in plaque size increase showed that the seasonal and geographical origin and thus evolutionary history are important drivers for the ecology of infections. The differences in plaque size change and when the plaques appear can be used to identify several aspects of the infections: (i) the later a plaque becomes visible, the potentially longer the latency period from the start of infections is; (ii) the faster a plaque increases, the faster the virus spreads; and (iii) the larger the plaque becomes, the stronger the infection is. However, liquid infection assays where host and virus traits are usually identified are needed to validate these assumptions. All of the mentioned characteristics of the infection will affect the infection dynamics within the ecosystem and potential effects on the *Ostreococcus* populations [50]. Therefore, future studies should consider incorporating and experimenting on the documentation of these infection attributes to better characterize and identify infection patterns related to the seasonal and geographical origin of both the hosts and viruses.

### Supplementary Information

Below is the link to the electronic supplementary material.

Supplementary file1 (DOCX 1315 KB)

## Data Availability

Data will be made accessible in Zenodo. Sequence data will be available in GenBank.
