# Supplementary material for: Seasonality and Strain Specificity Drive Rapid Co-evolution in an Ostreococcus-Virus System from the Western Baltic Sea
Source: Microb Ecol. 2023 Jun 3;86(4):2414–23. doi: 10.1007/s00248-023-02243-5 (PMC10640450; doi:10.1007/s00248-023-02243-5)
Supplement: Supplementary file 1 — Supplementary file1 (DOCX 1315 KB) [file 248_2023_2243_MOESM1_ESM.docx]

Supplementary material to Manuscript :

Seasonality and strain specificity drive rapid co-evolution in a Ostreococcus-virus system from the Western Baltic Sea.Luisa Listmann^1,2^, Carina Peters^1,2^, Janina Rahlff^3#^, Sarah P. Esser^4^, Elisa Schaum^1,2^

^1^ University of Hamburg, Institute for Marine Ecosystem and Fisheries Science, Olbersweg 24, 22767 Hamburg

^2^ Centre for Earth System Science and Sustainability, 20146 Hamburg

corresponding author: [luisa.listmann@uni-hamburg.de](mailto:luisa.listmann@uni-hamburg.de)

*Culturing condition of Ostreococcus cultures*

The *Ostreococcus* cultures were kept in f/2 media prepared with artificial seawater after Guillard, (1975), see below. The Baltic Sea is characterized by a strong salinity gradient ranging from ca. 8 in the East to ca. 20 in the West. For the culturing conditions, we decided to maintain the salinity conditions for the *Ostreococcus* at the level of *in situ* conditions (see Table S1 for details). Temperature also varied between the geographical regions [1] but even more between seasons. To make culturing and growth possible under laboratory conditions, we acclimatized the cultures to be grown at 18 °C, which represents an average temperature that *Ostreococcus* from all geographical regions experience and allows for enough growth and biomass production. The light conditions were set to 150 µE at a 12:12 light:dark cycle.

Artificial seawater was prepared using MilliQ water and adding sea salt to obtain the target salinity. Then the water was sterile filtered at 0.2 µm (Sartorius ®) and subsequently autoclaved. After cooling, nitrate and phosphate were added to a final concentration of 882 µmol L^-1^ and 36 µmol L^-1^, respectively. Trace metals and vitamins were added according to Guillard (1975).

**Table S1:** *Ostreococcus* (*O. tauri* and *O. mediterraneus*) strains and culturing conditions for husbandry in our laboratory

*Flow Cytometry*

*Ostreococcus* cells were counted at a rate of 65 µl min^-1^ on a flow cytometer (Accuri BD C6 Plus) using gating of cells characterized by the height of forward scatter and red fluorescence signal (Fig. S2). The forward scatter was set to a threshold of ca. 200 that allowed us to determine debris and potentially high amounts of bacteria in the experimental cultures. *Ostreococcus* is large enough to be well distinguished from bacterial cells or cell debris (Fig. S2). To count viral particles via flow cytometry we adapted the protocol by Brussaard, Corina, (2004) the following way: virus samples (diluted or undiluted) were combined with Glutaraldehyde (provider) to a final concentration of 1 % and after 15 min. of incubation at room temperature, frozen at -80 °C for later analysis. After quick defrosting at 37 °C, 40 µL of virus sample was combined with 960 µL of a mix of 0.02 µM filtered (Whatman Anotop ®) Tris-EDTA (Sigma Aldrich ®) buffer and SybrGold (Thermofisher ®) dye (2.5 µL 1´000x SybrGold per 1 mL TE buffer). The mixture was then heated to 80 °C for 10 min. and cooled at room temperature for another 15 min. After cooling, virus particles were counted at a rate of 17 µL min^-1^ in the flow cytometer. The FITC channel for green fluorescence was set to 20 and the virus populations identified using the side scatter and FIT-C signal (Fig. S2).

1. Santelia ME, Listmann L, Schnell S, Schaum E (2022) Predicting the unpredictable: heatwaves and history of variability shape phytoplankton community thermal responses within one generation. bioRxiv 2022.07.20.500758. https://doi.org/10.1101/2022.07.20.500758

2. Brussaard, Corina P. (2004) Optimization of procedures for counting viruses by flow cytometry. Appl Environ Microbiol 70:1506–1513. https://doi.org/10.1128/AEM.70.3.1506

3. Flores CO, Meyer JR, Valverde S, et al (2011) Statistical structure of host-phage interactions. Proc Natl Acad Sci U S A 108:. https://doi.org/10.1073/pnas.1101595108

Supplementary Figures


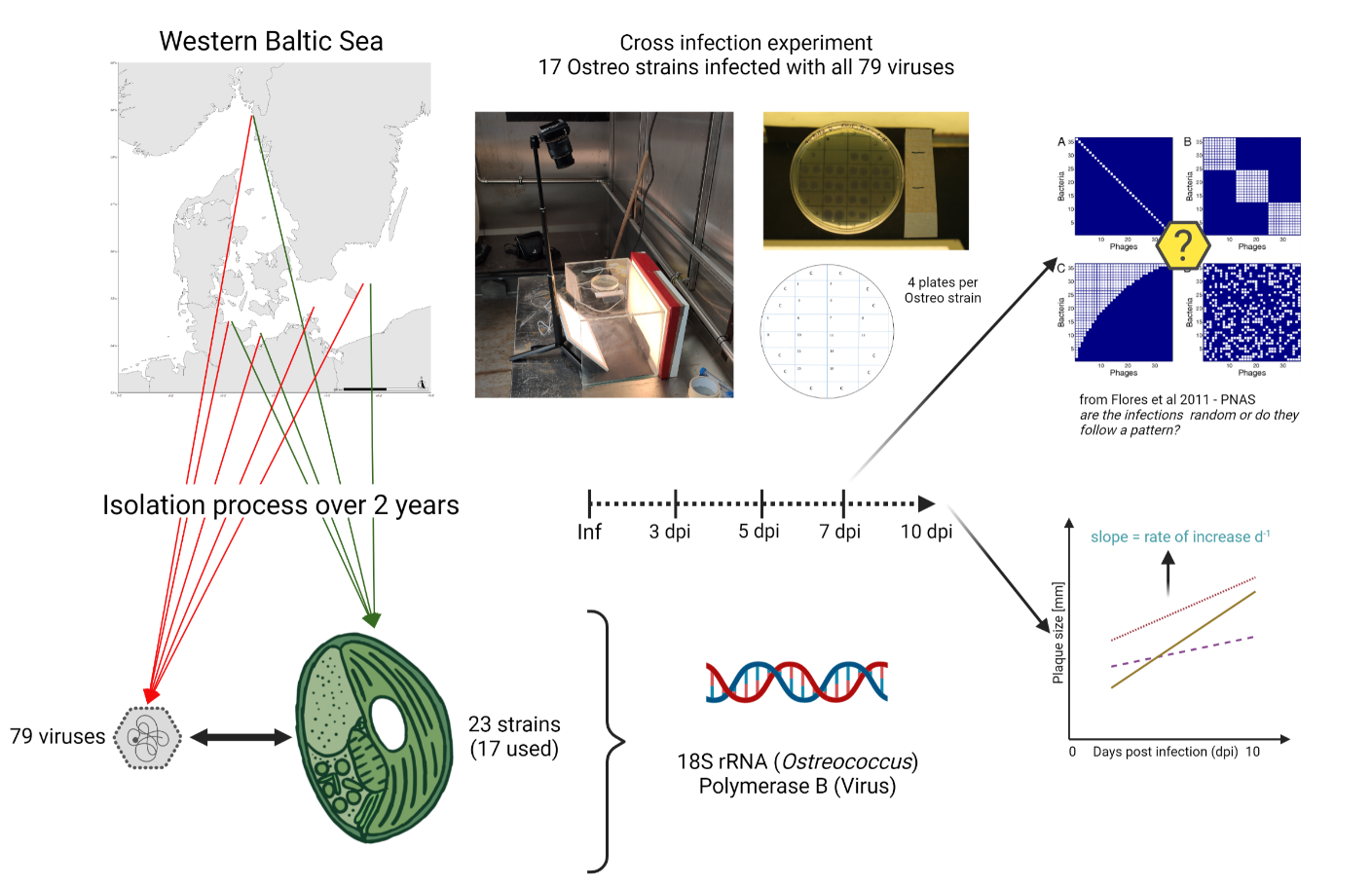
 **Figure S1.** The isolation procedure and set up of the cross-infection analysis as well as the genetic identification of both hosts and viruses is shown here in the left and middle section. The modularity analysis (example results [3] for these analyses are shown in upper right section) was done 7 days post infection (dpi), i.e. the latest time point when infections appeared, whereas the calculation of the plaque size changes was done over four time points post infection (3, 5, 7 and 10 dpi) (example slopes for plaque size increase are shown in lower right corner). Figure was created using the BioRender web application.


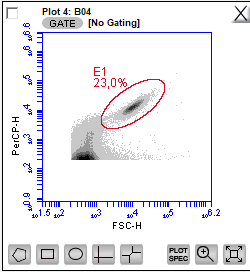

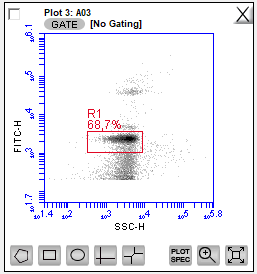


**Figure S2**: Representative flow Cytometry plot showing *Ostreoccocus* (left panel) and virus populations (right panel). Left plot: the x-axis depicts the forward scatter which is a proxy for size and the y-axis depicts the blue fluorescence which is an indicator for chlorophyll a content. Right plot: the x-axis depicts the side scatter which is a measure for granularity and can also be used as a proxy for size and the y-axis depicts the green fluorescence for anything dyed with SybrGold.

**Figure S3.** The first panel shows all *Ostreococcus* strains from which we started the isolation process for viruses. On the y-axis the *Ostreococcus* strains are listed with the following specifications: species (Ot= *Ostreococcus tauri*, Om= *Ostreococcus mediterraneus*), geographical and seasonal origin. The colours refer to the geographical origin and are the same again for the origin of the water samples for the virus isolation. On the x-axis all the water samples that were taken on the different cruises are listed in chronological (2019-2020) and geographical (West to East) order. In the top panel, the grey boxes indicate a combination of *Ostreococcus* strain with water sample for unsuccessful lysis, whereas green boxes indicate a successful lysis. Lower panel shows all remaining water and *Ostreococcus* combinations from which virus lysates were produced from plaque assays and that were used for the experiments later on.


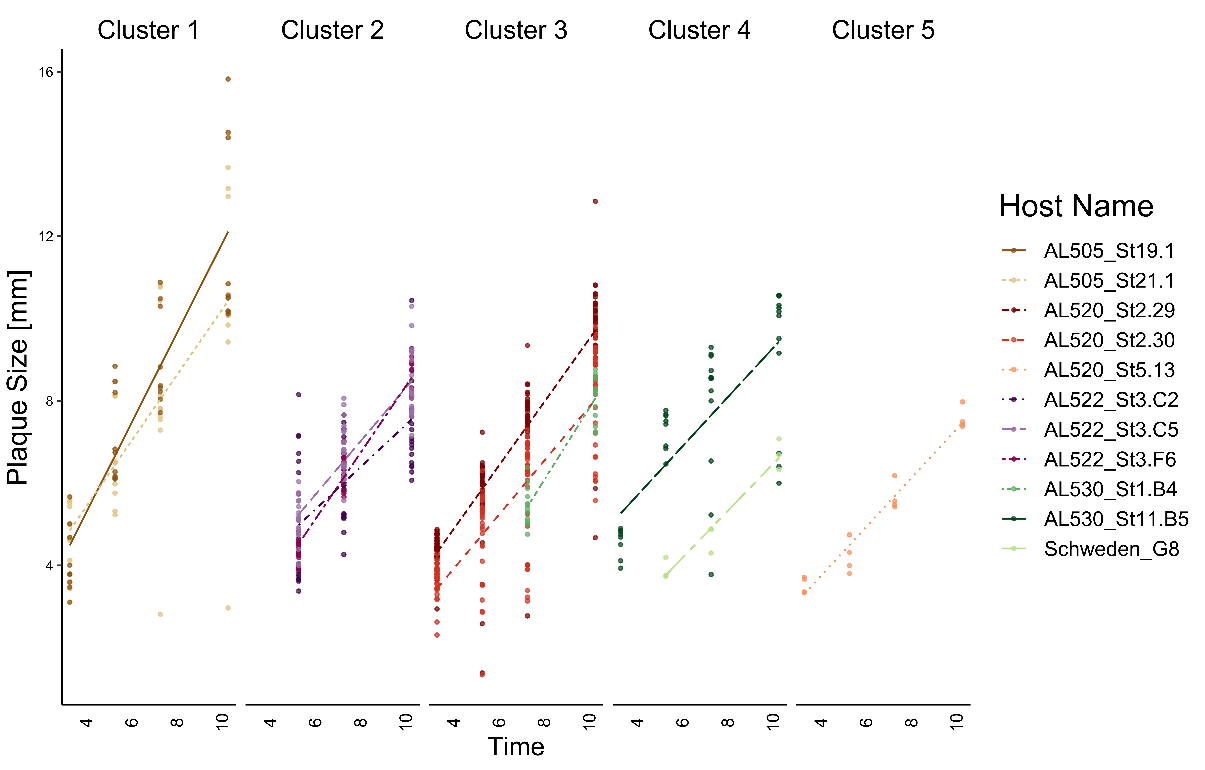


**Figure S4.** Points represent the plaque sizes in mm of the infections. Lines represent the linear regression fits for each host strain. The line types refer to the different hosts. The slopes indicate the rates of plaque size increase. Each panel shows the groups of infections belonging to one of the clusters in the infection matrix (infections per cluster range between 4 and 80 single infections.


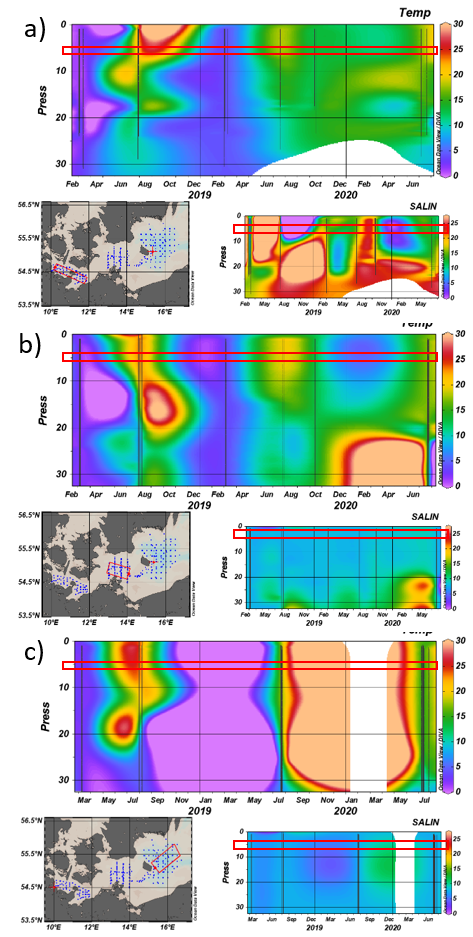


**Figure S5.** The figure shows the changes in temperature (bigger plot) and salinity (smaller plot) during the time of the sampling cruises. The different panels show the different research areas: Kiel Bight and Mecklenburg Basin KB and MB (a), Arkona Basin AB (b) and Bornholm Basin BB (c). The y-axes of the plots indicate the depth of the water, x-axes show the time of year during which the cruises took place, and the colors indicate temperature °C and salinity, respectively. In all basins, temperature changes throughout the year and according to seasons, whereas salinity is rather constant over time and differs between the different geographical regions.
